# Prevalence of Non-alcoholic Fatty Liver Disease (NAFLD) Among Diabetic Mellitus Patients in Saudi Arabia: Systematic Review and Meta-Analysis

**DOI:** 10.7759/cureus.51092

**Published:** 2023-12-25

**Authors:** Abdulrahman M Albeshry, Mohammed Abdulrahman Alasmari, Jaber Abdullah Alshahrani, Alaa Mohammed Alshahrani, Abdulmajeed Saad Almusma, Mohammed A Alfaya, Ali J Alfaifi, Mastoor A Alshahrani, Hamad Khulaif D Alharbi, Ali S Ali Etwdi, Eyad Aldawsari, Sundus Mohammad Zakir Hiyat Moazam, Mohammed Alshaiban, Saad N Al-Harthi

**Affiliations:** 1 Family and Community Medicine, Faculty of Medicine, University of Jeddah, Jeddah, SAU; 2 Medicine, King Khalid University, Abha, SAU; 3 Family Medicine and Medical Education, Armed Forces Hospital Southern Region, Khamis Mushit, SAU; 4 Family Medicine, Armed Forces Hospital Southern Region, Khamis Mushit, SAU; 5 Family and Community Medicine, King Khalid University, Abha, SAU; 6 Family Medicine, Primary Health Care Corporation (PHCC) Khamis Mushait Sector, Ministry of Health, Khamis Mushit, SAU; 7 Medicine, Al Jouf University, Sakaka, SAU; 8 Laboratory, Armed Forces Hospital Southern Region, Khamis Mushit, SAU; 9 Medicine, King Saud Bin Abdulaziz University for Health Sciences, Riyadh, SAU; 10 Family Medicine, Alhaqbani Medical Complex, Khamis Mushait, SAU; 11 Medicine, Najran University, Najran, SAU; 12 Medicine, King Faisal University, Dammam, SAU

**Keywords:** dm, diabetes type 2, nafld, systematic review and meta-analysis, fatty liver disease, non-alcoholic, prevalence

## Abstract

Non-alcoholic fatty liver disease (NAFLD) is a burgeoning global health concern, closely associated with the rising prevalence of type 2 diabetes mellitus (T2DM) and obesity. This systematic review and meta-analysis aim to comprehensively evaluate the prevalence of NAFLD in DM patients in Saudi Arabia, a country undergoing rapid socioeconomic changes. Our multifaceted search strategy identified four high-quality studies conducted between 2003 and 2022, covering hospital and community settings. The aggregate prevalence rate of NAFLD in DM patients was notably high, ranging from 47.8% to 72.8%. However, substantial heterogeneity (I² = 90.6%) was observed, indicating variability attributed to diverse study characteristics. The uniform application of ultrasound for diagnosis was noteworthy but raised concerns regarding sensitivity. This analysis underscores the urgency of public health measures for early detection and management of NAFLD in DM-prone populations in Saudi Arabia.

## Introduction and background

Non-alcoholic fatty liver disease (NAFLD), a condition characterized by excessive fat accumulation in the liver in the absence of significant alcohol consumption, has emerged as the most common liver disorder worldwide [1]. Its increasing prevalence is tightly associated with the rise in metabolic syndrome components, notably obesity and type 2 diabetes mellitus (T2DM) [2]. Saudi Arabia, a nation experiencing rapid socio-economic transformations, sees an escalating incidence of metabolic disorders due to changing lifestyles and urbanization, making the investigation of NAFLD within this setting highly relevant [3].

NAFLD represents a range of liver conditions, from basic steatosis to non-alcoholic steatohepatitis (NASH), potentially leading to severe complications such as cirrhosis and hepatocellular carcinoma [4]. Worldwide, the prevalence of NAFLD has risen from 25.3% in the period 1990-2006 to 38.0% in 2016-2019, with regional disparities mirroring demographic and lifestyle influences [5,6]. In Saudi Arabia, the scenario is intensified by prevalent obesity and T2DM, crucial factors contributing to NAFLD. Examining the prevalence and traits of NAFLD among the Saudi population is vital for crafting targeted health interventions and policies.

T2DM, a chronic metabolic disorder characterized by insulin resistance or deficiency, leading to elevated blood glucose levels, is a significant global health issue and a standalone risk factor for NAFLD [2]. The interrelation between T2DM and NAFLD is complex and reciprocal; not only does T2DM heighten the risk of developing NAFLD, but it also exacerbates glycemic management in diabetic individuals [7]. This dual challenge is particularly acute in Saudi Arabia, which records one of the highest T2DM rates worldwide.

Saudi Arabia's rapid socio-economic advancement in recent years has led to profound lifestyle shifts [8]. Urbanization, dietary changes, and increased sedentary habits have escalated obesity, T2DM, and, consequently, NAFLD rates [9-15]. These health issues significantly strain the healthcare system, emphasizing the necessity for a thorough epidemiological understanding within the Saudi Arabian setting.

This systematic review and meta-analysis address this need, focusing on the diagnostic challenges of NAFLD, especially in T2DM patients. Diagnosing NAFLD, particularly in its initial stages, poses significant complexities. This review investigates various diagnostic tools used in the selected studies; however, it is evident that ultrasonography (US) emerged as the most significant and widely utilized method, thereby being the primary focus, along with liver biopsy and non-invasive biomarkers, in assessing their influence on the reported prevalence of NAFLD.

Besides its epidemiological scope, this review also highlights the impact of different diagnostic techniques on prevalence figures, offering essential insights for healthcare practitioners and policy-makers in devising effective strategies for the prevention, early detection, and management of NAFLD in T2DM patients.

## Review

Materials and methods

Search Strategy

To systematically capture studies pertinent to our research question, we employed a multifaceted search strategy. This involved querying major databases, including PubMed, Scopus, Web of Science, and the Cochrane Library. Our search spanned literature published up to 2022, ensuring the inclusion of the most recent studies. We constructed a search algorithm using a combination of Medical Subject Headings (MeSH) terms and free-text words to maximize the retrieval of relevant articles. The primary search terms included “non-alcoholic fatty liver disease,” “NAFLD,” “diabetes mellitus,” “DM,” and “Saudi Arabia.” These terms were combined using the Boolean operators “AND” and “OR” to encompass related keywords and phrases. For example, our search string in PubMed looked like this: (“non-alcoholic fatty liver disease” OR “NAFLD”) AND (“diabetes mellitus” OR “DM”) AND “Saudi Arabia.”

To capture a broader spectrum of relevant literature, we expanded our search to include regional databases such as the Saudi Medical Literature Database and the Index Medicus for the Eastern Mediterranean Region. We also manually searched the reference lists of identified articles and relevant reviews for additional studies that might have been missed in the database search.

Inclusion and Exclusion Criteria

Our systematic review was inclusive of a broad spectrum of studies shedding light on the prevalence of NAFLD in DM patients within Saudi Arabia. We considered observational studies of various designs - cross-sectional, cohort, case-control, and both prospective and retrospective studies - involving DM-diagnosed patients. The prerequisite for inclusion was the use of established diagnostic approaches for NAFLD, ranging from liver biopsy and abdominal ultrasound to MRI, CT scans, liver function tests, and specific blood-based markers. Our objective was to collate data from diverse settings, both hospital and community-based, with no constraints on publication date or language, translating non-English studies for comprehensive analysis.

On the flip side, our exclusion criteria were set to uphold the scientific validity of our review. We disregarded studies reliant solely on self-reported NAFLD diagnoses, lacking clinical or laboratory corroboration. We also excluded research centered on fatty liver due to alternate causes, including alcohol consumption, drug-induced liver damage, and viral hepatitis. Single-gender studies were omitted to maintain gender parity in our analysis. Moreover, studies missing crucial details such as sample size, diagnostic approaches, or prevalence rates, which remained unobtainable from authors, were excluded. Lastly, non-peer-reviewed materials such as grey literature and editorials were also excluded to ensure data integrity.

Study Selection

Our selection methodology for studies was rigorously structured to ensure exhaustive identification and evaluation. Initially, all identified articles were imported into EndNote, facilitating duplicate removal. The first screening phase involved two independent reviewers meticulously assessing study titles to eliminate irrelevant or non-compliant studies. This was followed by a detailed review of abstracts by these reviewers to refine our selection further.

Articles deemed relevant at this stage underwent a full-text review by another pair of independent reviewers to confirm eligibility against our criteria. This comprehensive evaluation ensured a thorough assessment of methodologies, populations, and outcomes. Discrepancies at any stage were resolved through discussion, or by consulting a third reviewer for a conclusive decision. Additionally, a manual search of references in selected studies was conducted to ensure the inclusion of all relevant literature.

Data Collection

Our data extraction process was executed with meticulous precision and systematic rigor. Two independent reviewers were entrusted with this task, using a bespoke data extraction form designed for this review. This form captured key data points, including study characteristics, design, setting, population demographics, diagnostic methods for NAFLD, and main findings. In cases of data extraction variances, reviewers convened for consensus discussions, with a third reviewer providing an objective decision when necessary. This step ensured data collection accuracy and integrity.

Quality Assessment

In our systematic review, the quality assessment of the included studies was an integral part of the methodology, aimed at evaluating the methodological rigor and potential biases in each study. To this end, we adopted a well-established quality assessment tool tailored to observational studies. For this purpose, the Newcastle-Ottawa Scale (NOS) was chosen due to its comprehensive criteria that assess three broad perspectives: selection of the study groups, comparability of the groups, and ascertainment of the outcome of interest for case-control or cohort studies [11].

Each study was independently evaluated by two reviewers. They assessed the studies against the NOS criteria, which involve assigning scores based on the quality of selection, comparability, and exposure (for case-control studies) or outcome (for cohort studies). The scores were then used to categorize studies into three quality tiers: high, moderate, and low. High-quality studies were those that met most of the NOS criteria, indicating a lower risk of bias. Moderate-quality studies partially met these criteria, while low-quality studies had several unmet criteria, suggesting a higher risk of bias. The independent assessments by each reviewer were crucial in ensuring an unbiased and thorough evaluation. Any disagreements between the reviewers regarding the quality scores were resolved through a discussion to reach a consensus.

Statistical Analysis

In managing and analyzing data for this study on the prevalence of NAFLD in DM patients in Saudi Arabia, we utilized Rayyan (Rayyan Systems Inc., Cambridge, MA) - Qatar Computing Research Institute (QCRI) for effective article management [12]. This approach ensured the organized handling and selection of relevant studies. The statistical analysis was conducted using Stata version 17 (StataCorp LP, College Station, TX), chosen for its advanced capabilities in handling complex meta-analytical data [13]. Our meta-analysis specifically focused on the prevalence of NAFLD among DM patients, employing a random-effects model based on the weighted inverse variance method by Borenstein et al. [13]. This model is adept at accommodating the expected high heterogeneity due to variations in study designs, populations, and diagnostic criteria. We presented our results using forest plots, illustrating pooled proportions and 95% confidence intervals (CIs) for clarity and ease of interpretation.

To assess the degree of variability and robustness of our findings, we utilized Higgin’s I² test statistic, considering an I² value greater than 50% as indicative of high heterogeneity. This was complemented by sensitivity analyses, where individual studies were systematically excluded to evaluate their impact on the overall results, ensuring the stability and reliability of our findings. Additionally, we conducted subgroup analyses to explore prevalence variations among different DM patient populations. The significance of the results was determined using a p-value threshold of less than 0.05.

Results

Article Screening and Selection

**Figure 1:**
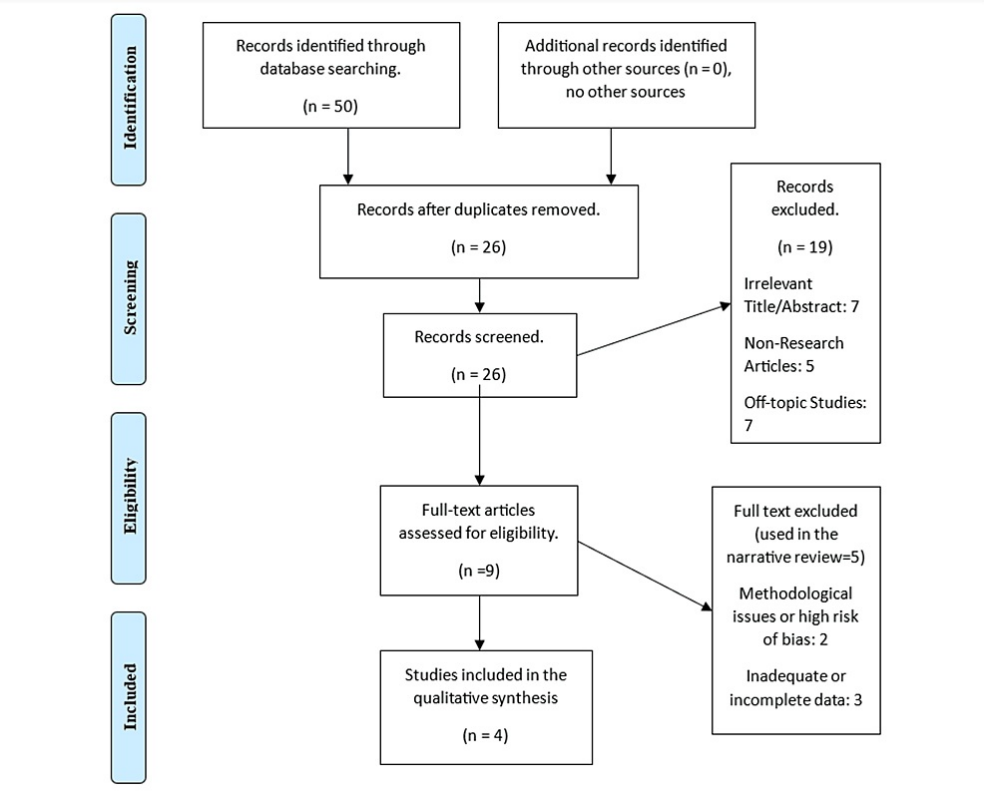
Flow diagram for the article screening and selection process of the studies

In the initial phase of our systematic review, a comprehensive search yielded 50 potential articles. Following deduplication, 26 unique articles remained. Rigorous screening excluded 19 articles for reasons such as irrelevance, non-research content, or deviation from the topic. A thorough full-text review of the remaining nine articles was conducted, leading to the selection of four studies meeting strict inclusion criteria for quality and relevance in our meta-analysis. The Preferred Reporting Items for Systematic Reviews and Meta-Analyses (PRISMA) [14] guidelines were employed, with its statement depicted in Figure 1.

NAFLD Prevalence in DM Patients in KSA

**Figure 2:**
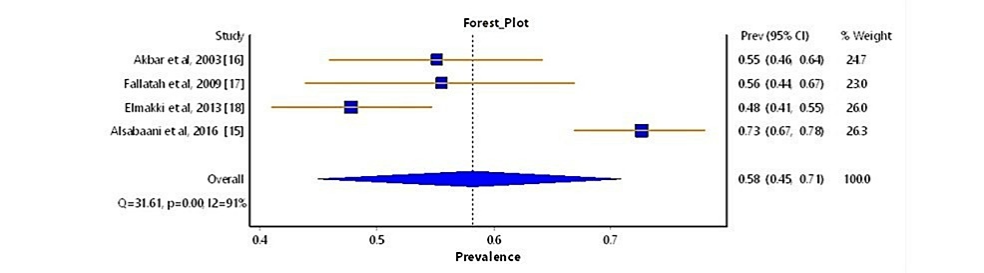
Forest plot of the prevalence of NAFLD in DM The plot displays prevalence rates of non-alcoholic fatty liver disease (NAFLD) among patients with diabetes mellitus (DM). Black squares indicate the prevalence from individual studies, sized according to study weight. Horizontal lines show confidence intervals, and the diamond represents the pooled prevalence and overall heterogeneity.

In our meta-analysis, we synthesized data from four separate studies carried out between 2003 and 2022, covering both hospital and community settings. These studies offered a detailed look at the prevalence of NAFLD in DM patients across Saudi Arabia [15-18]. A forest plot, shown in Figure 2, illustrates the individual and overall prevalence rates of NAFLD from each study, represented as point estimates with 95% CIs. These rates varied, ranging from 47.8% in the study by Elmakki et al. [18] to 72.8% in the study by Alsabaani et al. [15], demonstrating a notable range in the findings. The aggregate prevalence rate, determined through the common-effect inverse-variance model, was markedly high. The confidence interval underscored the precision and statistical significance of this combined estimate.

However, the heterogeneity across these studies was significant, as indicated by an I^2^ value of 91% and Cochran's Q statistic of 31.61 (p < 0.001), with a Tau² value of 0.063, suggesting substantial variability in NAFLD prevalence across different populations and study designs.

Characteristics of the Included Studies

The four studies included in our analysis provide a wide-ranging perspective on the prevalence of NAFLD in T2DM patients in Saudi Arabia, as detailed in Table 1. Conducted from 2003 to 2022, these studies include both cross-sectional and cohort designs and span hospital and community environments. The sample sizes ranged from 72 to 245 participants, with varied demographics in terms of gender and age. All studies used US for NAFLD diagnosis, with prevalence rates showing variability, the highest being 72.8%, as reported by Alsabaani et al. [15]. The diversity in these studies contributes significantly to a comprehensive understanding of NAFLD in this demographic.

**Table 1:** Characteristics of the included studies

Author	Study Type	Study Setting	Population	Participants	Males (%)	Age (years)	Diagnostic Method	Prevalence (95% CI)
Akbar et al., 2003 [16]	Cross-sectional	Hospital-based	T2DM patients	116	28.0	54 ± 12.8	US	55% (46.1-63.9)
Fallatah et al., 2009 [17]	Cohort study	Hospital-based	T2DM patients	72	41.7	58.5	US	55.6% (44.1-66.5)
Elmakki et al., 2013 [18]	Cross-sectional	Hospital-based	T2DM patients	207	54.1	-	US	47.8% (41.1-54.6)
Alsabaani et al., 2016 [15]	Cross-sectional	Community-based	T2DM patients	245	66.1	57.1 ± 13.5	US	72.8% (66.6-78.1)

Quality Assessment of the Included Studies

The methodological quality of the included studies was thoroughly evaluated, assessing aspects such as selection, comparability, and outcome measurement. Studies by Akbar et al. [16], Fallatah et al. [17], Elmakki et al. [18], and Alsabaani et al. [15] were classified as high-quality, each scoring between 7 and 8 out of a maximum of 9 points [15-17,19]. This assessment, outlined in Table 2, confirms the dependability of our findings and solidifies the strength of our systematic review and meta-analysis.

**Table 2:** Quality assessment of the included studies

Study (Author, Year)	Selection (Max 4 Points)	Comparability (Max 2 Points)	Exposure/Outcome (Max 3 Points)	Total Score (Max 9 Points)	Quality Tier
Akbar et al., 2003 [16]	3	2	2	7	High Quality
Fallatah et al., 2009 [17]	4	2	2	8	High Quality
Elmakki et al., 2013 [18]	3	2	2	7	High Quality
Alsabaani et al., 2016 [15]	4	2	2	8	High Quality

Discussion

In this systematic review and meta-analysis, we have undertaken a detailed exploration of the prevalence of NAFLD among DM patients in Saudi Arabia. By scrutinizing four rigorously selected studies, our research casts a spotlight on the burgeoning health issue of NAFLD, a matter of increasing concern in the wake of rising DM cases worldwide and, more specifically, within Saudi Arabia.

Our analysis reveals a strikingly high incidence of NAFLD in DM patients in Saudi Arabia, surpassing the prevalence rates observed in many other regions. These rates, ranging from 47.8% to 72.8%, not only illustrate the serious extent of the problem but also point to significant variability. This variability likely mirrors diverse study designs, diagnostic methods, and patient populations. Such a high prevalence is concerning and may be linked to genetic predispositions, dietary habits, and lifestyle practices prevalent in the Saudi population [20-22].

A noteworthy aspect of our analysis is the considerable heterogeneity (I² = 91%) observed across the studies. This suggests that the differences in prevalence rates are influenced by specific study characteristics, including demographic profiles, NAFLD diagnostic techniques, and research settings. This level of heterogeneity necessitates a careful interpretation of the pooled prevalence data, considering the unique contexts and methodologies of each study.

The exclusive reliance on US in all the reviewed studies is particularly significant. While US offers advantages such as being non-invasive, widely available, and cost-effective, its relatively lower sensitivity, particularly for mild NAFLD cases, raises concerns about potential underestimation of the actual prevalence in the diabetic cohort [23]. This underscores the need for incorporating more sensitive diagnostic methods, such as MRI or liver biopsies, in future studies to achieve a more accurate prevalence estimate.

Our study also highlights the multifaceted nature of NAFLD, shaped by genetic, environmental, and lifestyle factors [1,4,24]. The elevated incidence of NAFLD among DM patients in Saudi Arabia could partially be attributed to regional lifestyle factors, such as high-calorie diets and sedentary habits, which are known contributors to metabolic disorders [25]. These findings emphasize the need for targeted public health strategies focusing on dietary and lifestyle modifications to combat this trend. However, it also underscores a key limitation in our review: the heterogeneity in study designs and diagnostic methodologies.

The necessity for standardized diagnostic criteria and consistent study methodologies in NAFLD research emerges as a crucial theme from our review [26]. The current variation in diagnostic methods and study designs hinders direct comparisons between studies and calls for a standardized approach to ensure more precise and comparable data across different research settings [27]. Additionally, establishing standardized diagnostic criteria for NAFLD would facilitate more consistent and reliable prevalence estimates and allow for better comparisons across different studies and populations.

Considering the timespan of the studies, from 2003 to 2022, it is imperative to acknowledge the rapid lifestyle and healthcare changes in Saudi Arabia. These changes could influence the prevalence rates in more recent studies, highlighting the need for ongoing research and updated analyses for an accurate and current understanding of the NAFLD-DM interplay in this region.

One significant limitation of our review is the absence of general population studies focusing on NAFLD in Saudi Arabia. Our reliance on data primarily from specific subgroups, such as T2DM patients, limits our ability to generalize the findings to the broader population. This highlights a gap in the current literature and points to the need for future research encompassing a wider spectrum of the population.

Our review not only sheds light on the high prevalence of NAFLD among DM patients in Saudi Arabia but also underscores the urgent need for public health interventions. These interventions should focus on lifestyle modifications, early detection, and effective management of NAFLD and DM to reduce long-term complications such as cardiovascular diseases and liver complications. Furthermore, the review calls for future research directions, including more inclusive population studies and investigations into the progression rate of screen-detected NAFLD, especially in the context of T2DM. The increasing prevalence of NAFLD in Saudi Arabia demands an integrated approach, combining public health initiatives, research, and healthcare strategies, to effectively address this emerging health.

## Conclusions

In conclusion, this systematic review and meta-analysis have highlighted a significant prevalence of NAFLD in DM patients within Saudi Arabia. The findings underscore the complex nature of NAFLD in relation to DM, influenced by a myriad of factors, including lifestyle, genetics, and environment. The study calls attention to the necessity of robust public health strategies for the prevention and management of NAFLD, especially in high-risk populations. It also emphasizes the importance of ongoing research and the adoption of standardized diagnostic criteria to enhance our understanding and response to this growing health challenge.
